# The Combination of Electrochemistry and Microfluidic Technology in Drug Metabolism Studies

**DOI:** 10.1002/open.202200100

**Published:** 2022-09-27

**Authors:** Isobel Grint, Francesco Crea, Rafaela Vasiliadou

**Affiliations:** ^1^ School of Life, Health and Chemical Sciences The Open University Walton Hall, Karen Hills Milton Keynes MK7 6AA UK

**Keywords:** cancer. drug metabolism. electrochemistry, microfluidics, reactive Metabolites

## Abstract

Drugs are metabolized within the liver (pH 7.4) by phase I and phase II metabolism. During the process, reactive metabolites can be formed that react covalently with biomolecules and induce toxicity. Identifying and detecting reactive metabolites is an important part of drug development. Preclinical and clinical investigations are conducted to assess the toxicity and safety of a new drug candidate. Electrochemistry coupled to mass spectrometry is an ideal complementary technique to the current preclinical studies, a pure instrumental approach without any purification steps and tedious protocols. The combination of microfluidics with electrochemistry towards the mimicry of drug metabolism offers portability, low volume of reagents and faster reaction times. This review explores the development of microfluidic electrochemical cells for mimicking drug metabolism.

## Graphical abstract

**Electrochemistry** has the potential to compliment the current techniques, towards the mimicry of human drug metabolism. Usually is coupled to mass spectrometry for detection purposes and a metabolic profile is obtained in just a few minutes, without the use of animals or cells.

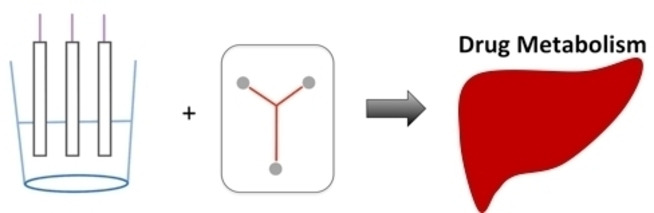

## 1 Introduction

Drug metabolism is a physiological process that occurs mainly in the liver.[^1^, ^2^, ^3^] During the process, exogenous compounds are broken down into smaller intermediates (metabolites) that eventually excrete from the human body via urine.^[4]^ Drug metabolism is divided into phase I and phase II (conjugation) metabolic reactions.^[5]^ Phase I reactions are catalysed predominantly by CYP‐450 enzymes, a protein superfamily found in the endoplasmic reticulum of hepatocytes.^[6]^ Phase I metabolism modifies the chemical structure of parent drug by oxidation, reduction or hydrolysis.[^7^, ^8^] Subsequently, the phase I metabolite serves as a substrate for phase II conjugation reactions. Phase II metabolism produces a more polar (water soluble) compound for excretion purposes.[^9^, ^10^, ^11^] Phase II reactions include methylation, glucuronidation, sulfation, conjugation with glutathione and amino acids.[^12^, ^13^, ^14^, ^15^, ^16^] Most of drugs are characterised by increased hydrophobicity,^[17]^ thus as expected drug metabolism plays a dominant role for their excretion. Particular attention should be driven on the generated metabolites and on how they might affect the normal function of human body.

**Figure 1:**
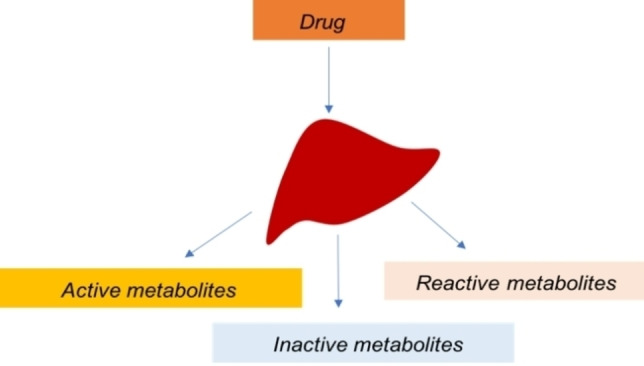
Different types of metabolites generated in drug metabolism.

Phase I and/or phase II reactions cause structure alternations to the substrate (drug) leading to three different types of metabolites. Depending on their chemical structure are classified into 1) Active metabolites, 2) Inactive metabolites and 3) Reactive metabolites, each category has a different activity and purpose in the human body, Figure 1. Active metabolites are very important in pharmacology, since they are capable to induce the desired therapeutic response. Sometimes, active metabolites are developed as drugs on their own and are available to the market^[18]^ targeting particular diseases. Inactive metabolites are intermediates without any therapeutic effect or significant pharmacological activity. Those intermediates are excreted from body and only the parent drug induces a therapeutic effect. The reactive metabolites are toxic intermediates and imply a major concern to the public health. They can cause unwanted side effects^[19]^ to human health, affecting both adults and children. Hepatotoxicity (Toxic hepatitis) is a common side effect of metabolite‐induced toxicity^[20]^ in humans.

Predicting the reactive metabolites is a crucial step in pharmaceutical research since unwanted side effects are avoided and safer drugs are produced for human use.^[5]^ Electrochemistry and particularly, the use of electrochemical cells has been proved as a powerful screening tool for identifying reactive metabolites. A pure instrumental set‐up involving the direct coupling of a cell with a suitable detection technique.^[21]^ Microfluidics, as miniaturised devices are capable to perform several analyses in an affordable fashion and have several applications in drug metabolism.^[22]^ The combination of electrochemistry with microfluidics offers low volume of reagents and faster metabolite synthesis, thereby bringing a new era into the field. In this review, we discuss the development of microfluidic electrochemical cells for mimicking drug metabolism. Considering that phase I and Phase II reactions require different chip designs and chemistry, their description has been divided into phase I and phase II metabolism. We also discuss about the chemistry of reactive metabolites, induced‐toxicity and cancer, as well as the potential of using electrochemistry as a complementary to mimic drug metabolism.

## 2 Reactive Metabolites, Toxicity and Cancer

Reactive metabolites are chemically active species formed during the phase I metabolism. Those intermediates are electron deficient molecules (electrophiles) that modify biomolecules (proteins and DNA) via covalent bonding and induce toxicity.^[23]^ Phase I metabolites are very unstable and short‐lived; thus, a trapping agent is required for their identification or detoxification.^[24]^ Glutathione (GSH) is a natural trapping agent for the detoxification of reactive phase I metabolites.[^13^, ^25^, ^26^, ^27^, ^28^, ^29^] GSH conjugation is an important phase II reaction catalysed by glutathione transferase enzymes.^[28]^ A detoxification pathway, in which the negatively charged GSH reacts with the positively charged phase I metabolite and forms a GSH‐electrophile conjugate.[^13^, ^30^] Covalent binding and toxicity are dose dependant, when GSH levels are depleted then the reactive metabolites can modify DNA and proteins.[^31^, ^32^, ^33^] Those reactions are known as type A adverse drug reactions and usually are avoided by dose adjustments.[^23^, ^34^] Acetaminophen is a well‐known example that can cause an overdose event and toxicity. The recommended dosage of acetaminophen is 650 mg to 1000 mg every 4 to 6 h, in adults and 15 mg/kg every 6 h, in children.^[35]^ Dosages differ significantly between adults and children considering differences in their anatomy and physiology.^[36]^ Higher amounts than the recommended can cause liver damage and hepatotoxicity. For example, acetaminophen is metabolised by CYP‐450 enzymes to reactive N‐acetyl‐p‐benzoquinone imine (NAPQI) and detoxified by GSH conjugation. However, in an overdose event, GSH levels are depleted and NAPQI binds to hepatic macromolecules causing irreversible hepatic necrosis.^[37]^ Type‐B (idiosyncratic) adverse drug reactions are dose depended only on susceptible patients and are identified after the drug is release to market.^[23]^

**Figure 2:**
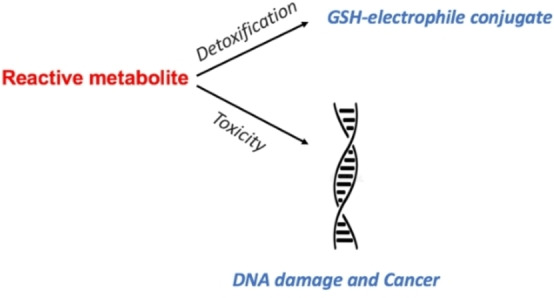
Reactive metabolism and cancer.

Cancer is one the of leading causes of death globally among adults and children.[^38^, ^39^, ^40^] Reactive metabolites are considered as chemical carcinogens, inducing mutations and deletions on genes, as well as alterations on genome transcription,^[41]^ Figure 2. They are activated usually during metabolism via oxidation, dealkylation and alkylation reactions to their carcinogenic forms.^[41]^

**Figure 3:**
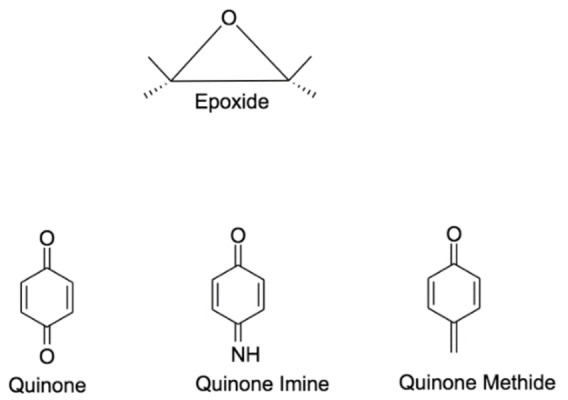
Important phase I reactive metabolites.

Reactive metabolites involve quinones, quinone imines, quinone methides and epoxides,[^23^, ^42^, ^43^] Figure 3. Free radicals also belong to the group of reactive metabolites due to their unpaired electron. Free radicals react with each other and form a covalent bond leading to new radical or a radical cation.[^21^, ^44^] Identifying the reactive metabolites during the early stages of drug development is a top priority for human health and research cost.^[5]^

## 3 Identifying Reactive Metabolites During Drug Development

**Scheme 1:**
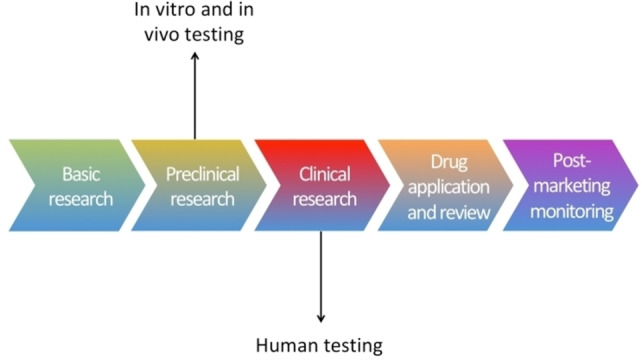
Steps of drug development.

Each year, billions of dollars are spent for the development of a new drug.[^45^, ^46^, ^47^] A tedious process requiring many years of investigations and trials.^[48]^ The stages of drug process and development involve 1) Basic research, 2) Preclinical research, 3) Clinical research, 4) Drug application and review and 5) Post‐marketing monitoring, Scheme 1.[^49^, ^50^] Drug metabolism is an important part of drug development since it determines the safety of a drug candidate. Reactive metabolites are identified and excluded from further studies saving time and costs.[^51^, ^52^] The safety and metabolism of a new drug candidate is investigated by in vitro (in glass) and in vivo (animal) studies during the preclinical research stages.[^53^, ^54^] There is not an ideal system that can mimic fully the human metabolism but rather a combination of in vitro and in vivo methods.^[55]^

Both in vitro and in vivo studies are important parts of preclinical research. They are used to predict metabolic pathways and metabolites prior to human testing.^[56]^ In vitro studies are always conducted first, as screening tools to characterize metabolites, rule out metabolic pathways and provide suggestions for the in vivo studies. In vitro metabolism includes liver microsomes, slices, hepatocytes, recombinant enzymes and S9 liver functions.[^57^, ^58^, ^59^, ^60^, ^61^] In vivo metabolism is focused on metabolic profiling in blood, urine and bile.^[62]^ Different species are used like rabbits, rats, mice, dogs, guinea pigs and monkeys.[^63^, ^64^] The advances in electrospray ionization (ESI) and atmospheric pressure chemical ionization (APCI) have prioritized the use of mass spectrometry (MS) on drug metabolism investigations.^[65]^ An excellent detection method which is coupled with liquid chromatography (LC) to provide both separation and detection. Liquid chromatography/mass spectrometry (LC‐MS) is a standard and approved method for investigations in drug metabolism.Nuclear magnetic resonance (NMR) is applied for structure confirmations and infrared spectroscopy (IR) for characterizing functional groups.[^64^, ^65^]

Analytical techniques are an ntegral part of both preclinical and clinical studies. After the completion of preclinical research, drug candidates are tested in humans. Clinical research is initially applied on healthy volunteers and gradually progresses to more targeted groups.^[66]^ Trials are conducted mostly on adults for ethical reasons since infants and children belong to vulnerable groups.^[67]^ Both preclinical and clinical research are vital towards the mimicry of drug metabolism and the identification of reactive metabolites.^[5]^ In vitro and in vivo methods, can reduce the toxic effects and decrease the chances of harmful drugs to reach clinical trials, saving time and costs. For those reasons, there is a constant need for new, affordable, automated and faster techniques capable to monitor metabolism of drug candidates.

## 4 Electrochemistry as a Complementary Technique in Preclinical Research

**Figure 4:**
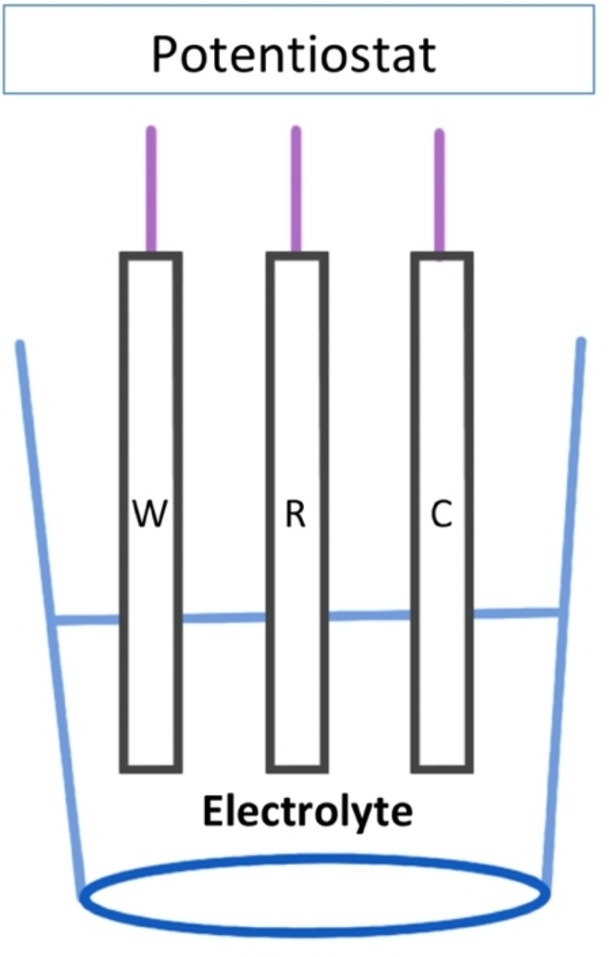
Electrochemical cell.

Electrochemistry is a branch of chemistry concerned with chemical and electrical phenomena. Electrochemical reactions can be oxidations or reductions, involving the transfer of electrons between a solid electrode and solution.[^68^, ^69^] Experiments are realised in an electrochemical cell with a three‐electrode configuration, a working electrode (W) in which the reaction takes place, a reference electrode (R) that provides a stable potential to the system^[70]^ and a counter electrode (C) that close the circuit and enables the charge to pass through the cell. The electrodes are immersed in a solution containing the compound of interest and supporting electrolyte. Potentiostat is an external device connected to electrochemical cell that controls the applied potentials on the working electrode,^[69]^ Figure 4.

The majority of phase I reactions are oxidations and reductions; thus, electrochemistry is ideal to simulate phase I metabolism.^[71]^ Phase II reactions are also mimicked by adding trapping agents on the electrochemical cell.^[72]^ GSH conjugation is the most popular phase II reaction in electrochemical metabolism. Electrochemical methods are coupled with a suitable detection technique such as MS and complement the current preclinical techniques, saving time and cost.[^71^, ^72^, ^73^] Conventional flow cells coupled to MS[^75^, ^76^, ^77^, ^78^, ^79^, ^80^] have been used for the mimicry of drug metabolism and simulated successfully a range of oxidation reactions such as dehydrogenation, aromatic hydroxylation, heteroatom oxidation and N‐dealkylation.[^81^, ^82^] Screen–printed electrodes have also been used for investigations in drug metabolism.[^83^, ^84^] These are miniaturised and disposable electrode systems operating at ul levels. The electrodes are fabricated from inks or paste and are capable to investigate a range of electrochemical parameters such as stability studies and synthesis. Screen‐printed electrodes offer low costs, portability, low volume of reagents, simplicity and integration on chip. The electrochemical synthesis of phase I metabolites is simple and requires both the optimization of potential and choice of appropriate electrode material.[^85^, ^86^, ^87^, ^88^] Carbon electrode is a standard choice for electrochemical metabolism but a diamond electrode can also be used to achieve higher potentials^[89]^ and more complicated reactions.

Generating electrochemically the metabolites is a very simple and effective approach. At the appropriate potentials, the desired metabolites are formed and a mass voltammogram is created, showing a range of applied potentials over the mass spectra of corresponding metabolites.^[90]^ The choice of buffer is critical and depends on the compatibility with MS. Phosphate buffers are avoided and alternatively ammonium acetate or ammonium formate are widely used for the hyphenation of electrochemistry with MS.^[75]^

Traditional methods are more complicated compared to electrochemical metabolism. They require several months of rigorous investigations and involve a number of sampling and extraction steps. Metabolites are isolated from biological samples, usually by solid phase extraction and then are ready for further investigations and detection purposes. However, in electrochemistry the metabolites are analysed directly without any sample preparation steps. A clean matrix is used, which is free from cells and proteins. Avoiding the purification step is one of the biggest advantages since it ensures higher metabolite yields and faster analysis.

## 5 Microfluidic Electrochemical Cells

Transferring electrochemical techniques on chip to generate reactive metabolites has become a very popular technique the last decade. The combination of electrochemistry with microfluidics is an affordable technology operating at extremely low volume of reagents. Microfluidic designs and specific set‐ups have been developed enabling the successful mimicry of both phase I and phase II metabolism.

### 5.1 Advantages of microfluidic electrochemical cells

The combination of electrochemistry with microfluidics, provides a pure instrumental approach that can be used as a complementary technique, over the current methods. The approach aims to reduce the unethical use of animal models, avoid the tedious extraction processes and reduce the overall costs. In vivo metabolism requires special facilities, all animals should be kept in a safe and clean environment, developed particularly for that application. Special training on how to handle and keep animals in captivity is essential and mandatory for all the working personnel. Furthermore, experiments on animals require ethical approvals prior to the start of any research work. Animal models are expensive to obtain and at the end of experiments, all animals are killed for ethical reasons. However, electrochemical methods don't require an extensive training or a licence to operate. It's easily and applicable from junior to senior lab members. The miniaturized format offers low volumes of solvents, reagents and wastes, thereby reducing the costs, save the environment and avoid unwanted contaminations. Also, the metabolites are generated and detected in just a few minutes.[^91^, ^92^] Furthermore, the online coupling of chip to MS provides an easy and automated procedure. As a consequence, microfluidic electrochemistry has potential applications during the early stages of drug development and discovery, where fast screening is highly required to select the best possible candidates and test compounds at low concentrations.[^93^, ^94^] Herein, we explore the development of the latest microfluidic electrochemical cells for phase I and phase II metabolism. Considering that different requirements are needed in terms of chip design, chip development and chemistry involved, the microfluidic electrochemical cells in this review are divided into phase I and phase II metabolism.

### 5.2 Microfluidic electrochemical cells for phase I metabolism

**Figure 5:**
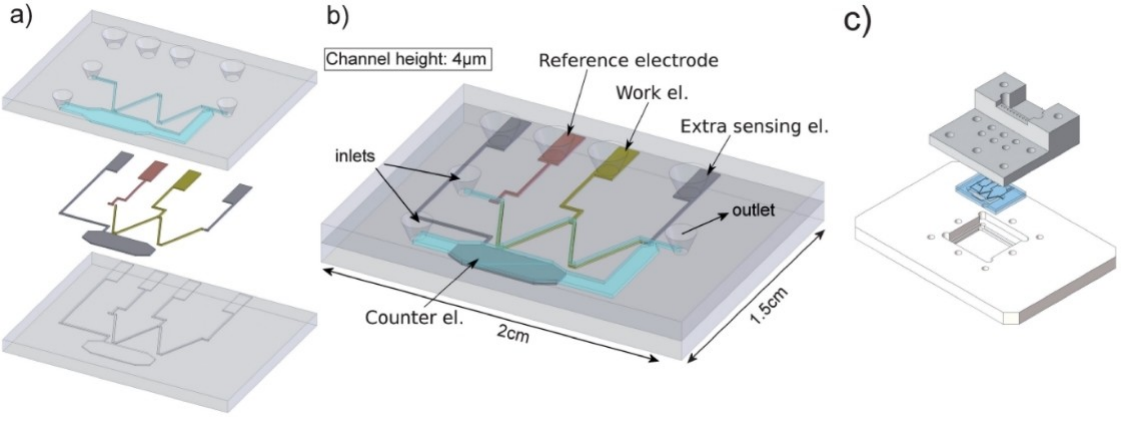
Microfluidic cell for mimicking phase I metabolism. The chip structures reproduced from Ref. [95] Copyright (2022), with permission from Royal Society of Chemistry.

A microfluidic electrochemical cell for specific applications in drug metabolism was developed by Odjik et al.,^[95]^ Figure 5. The chip simulated successfully the oxidative metabolism of amodiquinone, an anti‐malaria medication associated with some rare but serious side effects. At the optimized potential of 1000 mV, the drug metabolized into the corresponding phase I metabolites: Amodiaquine‐quinone imine (AQQI), N‐dehylated AQQI, N‐dehylated AQ and aldehyde. The flow rate was optimized at 1 μl min^−1^, ensuring 97 % of conversion rates. The main channel and electrode wells were at nL and nm levels respectively, enabling the use of low volume of reagents. The chip was fabricated with Pyrex glass and a three‐electrode system was incorporated, composed of a platinum working electrode, a platinum counter electrode and a pseudoreference palladium electrode. The counter electrode was placed in a side channel to prevent the formation of unwanted electrochemical products. The phase I metabolites were collected in sample loop and then introduced into a 6‐port valve for separation and detection purposes. The obtained metabolites were in an agreement with studies performed on a commercially available flow cell (Flexcell, Antec). However, the difference in volumes between the chip and sample loop causes limitations for time specific experiments.

Significant improvements on the reference electrode have increased the functionality of chip. A new version was developed by the same group.^[96]^ Particularly, the reference electrode was replaced with an iridium oxide film, offering greater stability and resistance to long term exposures in acidic and neutral solutions. Furthermore, higher potentials (1750 mV) were applied enabling the simulation of a range of oxidation reactions like N‐Dealkylation, dehydrogenation and oxygenation. The obtained electro‐generated phase I metabolites of procainamide were in a good agreement with experiments tested on animal and human cells.

Conventional rates, flow rates and compatibility of chip with ESI/MS are highly related parameters for a fully functional chip. As seen previously by Odjik et al. 2009,^[95]^ flow rate incompatibilities prevented the direct hyphenation of chip with the detection system. Particularly the microfluidic electrochemical cell was operating at low flow rates and the ESI/MS at much higher flow rates. For this reason, a six‐port valve was added to introduce the electro‐generated products on the separation and detection systems. However, the electro‐generated metabolites remained on the six –port valve for some time and this might lead to product degradation since phase I metabolites are extremely unstable. Direct hyphenation of chip with MS enables a more automated technique, simplicity of use and prevents any degradation of electro‐generated metabolites.

The addition of frit channels^[97]^ between the working electrode and the counter electrode was presented by the same group. In total two frit channels with a cross‐sectional area of 5 μm×100 μm and a length of 9.4 mm were integrated on chip, in an attempt to archive longer product diffusions between the platinum working electrode and platinum counter electrodes and thus to avoid any unwanted interferences. The pseudo‐reference electrode with cross sectional area of 500 μm×200 μm was placed at the bottom of the inlet channel, while the platinum working electrode and platinum counter electrode cross sectional areas of 5 μm×500 μm and length 24 mm, both were placed at the bottom of main channel. The novel chip permitted the application of higher flow rates up to 8 μl.min^−1^, allowing higher conversional rates. Furthermore, unwanted potential drops are prevented through the uniform distribution of current between the working electrode and counter electrode. An identical chip without frit channels was developed for comparison and evaluation purposes. The standard chip achieved a total conversion at 0.25 μl. min^−1^, which is impractical to coupled it directly with MS. As a consequence, the new design improved the functionality of microfluidic electrochemical cells integrated with metal electrodes. The focus of the particular investigation was to improve current distributions and flow rate and thus a simple metabolic pathway was simulated at 700 mV, involving the dehydrogenation of mitoxan.

Phase I metabolites such as quinones, radicals and quinone imines are characterized by instability and short half‐lives. Thereby, those metabolites are identified as GSH‐adducts on the in vitro and in vivo metabolism. However, in microfluidic electrochemistry/MS the integration of a miniaturized ESI needle with an electrochemical cell, reduced significantly the transition times between chip and MS.^[98]^ Short‐lived phase I metabolites were obtained on the mass spectra without the need of a trapping agent. An example of a pure electrochemical set up that can overcome limitations with traditional metabolism. The short‐lived metabolite chloropromazine radical travelled in just 4.5 seconds from chip to ESI/MS.Simple modifications including the addition of reaction coil permitted the phase II GSH metabolism. GSH was added on the effluent of working electrode and the reactive metabolite NAPQI was generated at 1200 mV. Both trapping agent and NAPQI reacted chemically for 3 mins in the reaction coil and APAP‐GSH adduct detected on MS. The same set‐up also mimicked the protein metabolism using carbonic anhydrase I, a protein target composed of several N nucleophilic sites. Protein‐adducts were formed in a same manner like GSH‐adducts.

### 5.3 Microfluidic electrochemical cells for phase II metabolism

**Figure 6:**
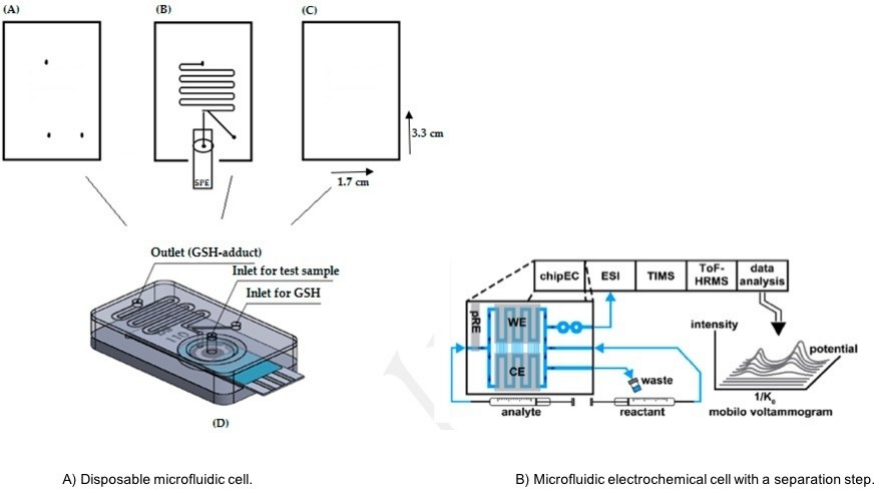
Microfluidic electrochemical cells for mimicking phase II metabolism. Chip structure A is reproduced from Ref. [99] under a CC BY licence. Chip structure B reproduced from Ref. [103] copyright (2022) with permission from ACS Publication.

Contamination and memory effects are widely seen on electrode materials. Strong adsorption phenomena enable the deposition of both reactants and products, on the surface of working electrode. This can possibly be more dominant over flow conditions, in which the working solution is constantly pumped on the electrode surface. A disposable and affordable microfluidic device^[99]^ was developed to overcome contamination issues, reduce further the costs and provide an easy and straight forwarded technique. A single use screen‐printed electrode was integrated with a serpentine reaction channel to mimic phase II metabolism, Figure 6A. The microfluidic device fabricated from three polycarbonate layers (3.3 cm and 11.7 cm length×width) with the electrodes and reaction channel placed on the middle layer. The total volume of the screen‐printed electrochemical cell was 32 μL. The electrode configuration composed of a carbon ink working electrode, a silver ink pseudo reference and carbon ink counter. The surface of the working electrode was larger compared to the counter electrode, preventing any interferences on product formation. Screen–printed electrodes can provide a plethora of information regarding the parent drug and generated metabolites. Several investigations were performed off chip using only screen‐printed electrodes. Important parameters such as metabolite stability, effect of pH and kinetics, provided a complete picture and supported the final product synthesis on chip.

The cost per chip was only five GBP and provided a proof of principle towards the mimicry of phase II metabolism, using affordable materials and electrodes. The methodology is very simple and can be performed by junior members in lab without any significant training or experience. The chip was coupled online to ESI/MS, the potential was applied from the potentiostat and the metabolites were formed in less than 10 minutes.

Phase I and phase II reactions of acetaminophen and dopamine were mimicked successfully inside the disposable device. The corresponding metabolites: GSH‐adducts, polymers and GSH disulphides were obtained on the mass spectra. The metabolites are in a good agreement with published data conducted in conventional metabolism studies.

Boron Doped Diamond (BDD) electrodes are excellent materials characterized by largest solvent working potential window, low background currents, resistance to fouling^[100]^ and capability to withstand extreme potentials.^[101]^ All those advantages make BDD electrodes exceptional materials for electrochemical metabolism on chip. However, those electrodes are expensive compared to standard carbon electrodes. The combination of BDD with a rotation mixer^[102]^ offered higher potentials and faster mixings between metabolites and trapping agents. Particularly, the design of mixer permitted very fast diffusions just in a few seconds, offering faster analysis times. The chip had four layers composed of 1) BDD insulator layer, 2) sputter platinum layer, 3) SU‐8 layer and 4) a borosilicate glass. All electrodes (working, reference and counter) were placed on the first insulator layer and the channels on the platinum layer. An identical chip using a platinum working electrode was fabricated for comparison reasons. 1‐hydroxypyrene was used as a test compound at 800 mV. A range of phase II metabolites such as GSH‐adducts and di‐GSH adducts were formed, as well as diols and quinone di‐adducts. Comparing the performance of platinum with BDD electrode, the former produced limited metabolites and lower intensities, confirming the excellent performance and stability of BDD electrodes on electrochemical metabolism.

Identifying isomeric metabolites is a real challenge in microfluidic electrochemistry, considering their instability and quick degradation. Owe karst team hyphenated online for the first time in electrochemical metabolism an electrochemical chip with ion mobility spectrometry (IMS),^[103]^ an analytical technique capable to separate compounds in 1 s. Traditional methods like HPLC require several minutes to separate the electro‐generated metabolites and this might cause degradation issues. However, separation columns have been added successfully on previous instrumental set‐ups involving conventional flow cells. Thus, further investigations in HPLC separation and microfluidic electrochemistry should be drawn to optimize conditions towards a faster analysis. In this investigation, an electrochemical cell was integrated with a mixer using paracetamol as model compound. The metabolites were separated online by IMS and detected online by MS. The particular set‐up provides a series of analytical steps performed in just a few minutes using extremely low volumes of reagents, Figure 6B.

Significant improvements have been reported the last years on microfluidic electrochemical cells, as summarized in Table 1. Different fabrication methods, materials and electrodes, as well as designs have been used to generate different metabolites and improve their performance.

**Table 1:** Description of microfluidic electrochemical cells.

Microfluidic Chip	Reactions	Electrode	Fabrication Material	Advantages	Reference
Glass chip	Phase I	Platinum	Borosilicate glass	− Low volume	95
Chip/iridium oxide film	Phase I	Platinum	Borosilicate glass	− High potentials	96
Chip with frit channels	Phase I	Platinum	Borosilicate glass	− High flow rate − Prevents potential drop	97
Chip/ESI needle	Phase I	Platinum	Borosilicate glass	− Short‐lived metabolites	98
Disposable Chip	Phase II	Carbon	Polycarbonate	− Single use − Low Cost	99
BDD electrode and rotation mixer on chip	Phase II	BDD	Borosilicate glass/ diamond and platinum layer	− High potentials − Faster diffusion	102
Chip/IMS/MS	Phase II	BDD	Borosilicate glass	− Separation	103

## 6 Future Directions and Perspectives

Different microfluidic electrochemical cells have been explored as tools towards the mimicry of drug metabolism. Most of the electrochemical cells were integrated with other processes to target specific metabolites, most of them highly reactive and short‐lived. The field is new, challenging and highly interdisciplinary combining both science and technology. Future directions should be concentrated on the development of a functional chip that can practically complement the current in vitro and in vivo methods focusing on reusability and mimicry in a range of metabolic reactions.

### 6.1 Reusability

Reusability is the most important parameter in the field, how many times a microfluidic electrochemical cell can be used, to simulate the same or different metabolic reactions. The reusability of chip is related to a range of factors such as fabrication materials, electrodes, chip design and cleaning procedures. Some cleaning procedures are destructive and might damage the chip after a few usages. There are numerous cleaning and activating protocols in literature^[104]^ that can be adapted in microfluidic set ups. However, a balance must be established between the quality of electro‐generated products and reusability. Disposable electrodes and materials must be considered for future chip designs, if the reusability of those devices is limited.

### 6.2 Metabolic reactions

The metabolic reactions that have been mimicked up to date are focused mainly on CYP‐450 enzymes (phase I). Metabolic reactions catalysed by other enzymes (both phase I and phase II) are currently missing from literature, creating a limitation towards the development of a fully functional chip.

## 7 Conclusions

Microfluidic electrochemical cells coupled to MS is a growing field with significant advances over the last decade. A wide range of parameters have been investigated involving different chip designs, disposable materials, different electrodes and instrumental set‐ups towards the mimicry of both phase I and phase II metabolism. A pure instrumental technique without the need of expensive enzymes, cells or animal models. The technique can be used as a primary screening tool to complement the traditional methods and has potential to be used as a future alternative for many reactions in pharmaceutical and medical research.

## Conflict of interest

The authors declare no conflict of interest.

8

## Biographical Information

*Isobel Grint is a Research Consultant currently working with the Open University, after having completed a research internship with them in 2021. She graduated from the Open University in 2020 with a Bachelor's degree in Natural Sciences specialising in Chemistry. She has been involved in research projects in multiple disciplines including biology, chemistry, micro‐engineering, and diversity & inclusion. She is interested in novel drug development methods, diversity & inclusion, and sustainability*.

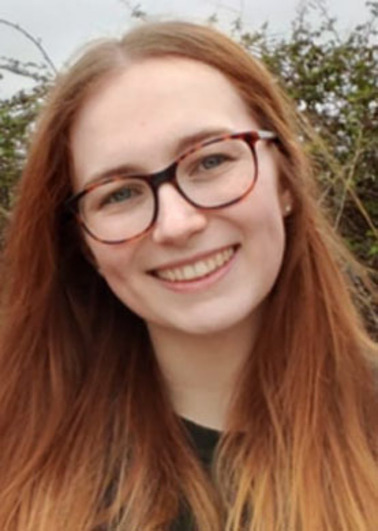

## Biographical Information

*Francesco Crea leads a lab investigating the role of non‐coding RNAs and epigenetics in advanced prostate cancer. He is Director of Research in the School of Life Health and Chemical Sciences at the Open University. He has received funds by Cancer Research UK and a Merit Award from the American Society of Clinical Oncology*.

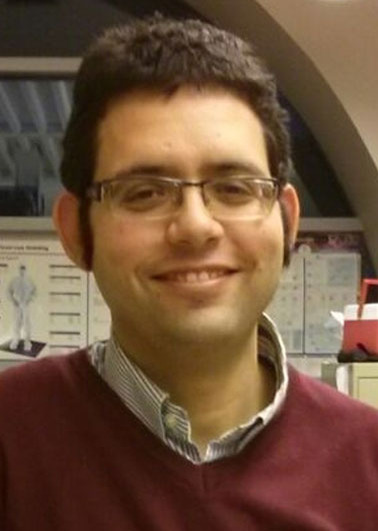

## Biographical Information

*Rafaela Vasiliadou received her Bachelor's degree in Human Biology, Master's degree in Analytical and Forensic Chemistry and PhD in Analytical Chemistry from the University of Hull (UK). She spent her post‐doctorate years in the group of Professor Nick Lane at University College London (UK). Then, she joined the School of Life, Health and Chemical Sciences at The Open University (UK), as a lecturer in Biological Chemistry. Her research is interdisciplinary focusing in Microfluidics, Electrochemistry/Mass Spectrometry Drug Metabolism and Proto‐metabolism*.

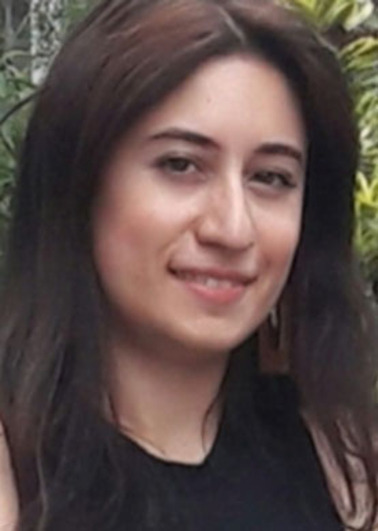

## Data Availability

Data sharing is not applicable to this article as no new data were created or analyzed in this study.
